# Determination of optimal daily light integral (DLI) for indoor cultivation of iceberg lettuce in an indigenous vertical hydroponic system

**DOI:** 10.1038/s41598-023-36997-2

**Published:** 2023-07-05

**Authors:** Kishor P. Gavhane, Murtaza Hasan, Dhirendra Kumar Singh, Soora Naresh Kumar, Rabi Narayan Sahoo, Wasi Alam

**Affiliations:** 1 Division of Agricultural Engineering, ICAR- Indian Agricultural Research Institute, New Delhi, India; 2 Centre for Protected Cultivation Technology, ICAR-Indian Agricultural Research Institute, New Delhi, India; 3 Division of Environment Science, ICAR-Indian Agricultural Research Institute, New Delhi, India; 4 Division of Agricultural Physics, ICAR-Indian Agricultural Research Institute, New Delhi, India; 5 ICAR-Indian Agricultural Statistics Research Institute, New Delhi, India

**Keywords:** Light responses, Plant physiology, Ecology, Physiology, Plant sciences, Environmental sciences, Energy science and technology

## Abstract

The indoor cultivation of lettuce in a vertical hydroponic system (VHS) under artificial lighting is an energy-intensive process incurring a high energy cost. This study determines the optimal daily light integral (DLI) as a function of photoperiod on the physiological, morphological, and nutritional parameters, as well as the resource use efficiency of iceberg lettuce (cv. Glendana) grown in an indoor VHS. Seedlings were grown in a photoperiod of 12 h, 16 h, and 20 h with a photosynthetic photon flux density (PPFD) of 200 µmol m^−2^ s^−1^ using white LED lights. The results obtained were compared with VHS without artificial lights inside the greenhouse. The DLI values for 12 h, 16 h, and 20 h were 8.64, 11.5, and 14.4 mol m^−2^ day^−1^, respectively. The shoot fresh weight at harvest increased from 275.5 to 393 g as the DLI increased from 8.64 to 11.5 mol m^−2^ day^−1^. DLI of 14.4 mol m^−2^ day^−1^ had a negative impact on fresh weight, dry weight, and leaf area. The transition from VHS without artificial lights to VHS with artificial lights resulted in a 60% increase in fresh weight. Significantly higher water use efficiency of 71 g FW/L and energy use efficiency of 206.31 g FW/kWh were observed under a DLI of 11.5 mol m^−2^ day^−1^. The study recommends an optimal DLI of 11.5 mol m^−2^ day^−1^ for iceberg lettuce grown in an indoor vertical hydroponic system.

## Introduction

The global population is expected to reach 9.7 billion by 2050, out of which 70 percent will live in urban areas^1^, which will put the urban infrastructure under stretch. By 2023, India is predicted to overtake China as the world's most populated country, with its population growing at the greatest rate between now and 2050^2,3^. The effect of climate change, depleting natural resources, and increased urbanization will lead to less land being available for farming. The effects of climate change on crop yields imply that yield reductions could reach up to 60% by the end of the century, depending on the type of crop, the region, and the future climate change scenario^4^. To tackle these challenges, modern cultivation techniques like vertical farming need to be practiced to ensure food security^5,6^. These systems can grow more plants per unit of area than the traditional cultivation methods, resulting in a higher crop production. This is accomplished by establishing crop production in the vertical dimension^7^. Vertical farming can be done in greenhouses and plant factories, two of the typical forms of controlled-environment agriculture^8,9^.

Lettuce is one of the most commercially significant crops grown around the world. Lettuce is the ideal crop for vertical farming because of its short growth cycle, low energy demand, quicker growth, low calorie count, and it also contains significant levels of fiber, minerals, and vitamins, in addition to beneficial bioactive substances^10^. Iceberg and Great Lakes lettuce are two of the most commonly cultivated lettuce varieties of head lettuce in India. After China and the USA, India is the third largest producer of lettuce in the world, accounting for 14% of the total sown area around the globe but producing only 4.1% of the total output^11^. Hence, there is growing need to increase the production and productivity of lettuce in India using modern intensive cultivation techniques like vertical farming.

Light is one of the most significant environmental variables in vertical farming that influences plant development and governs plant activity based on light quantity, quality, and direction^12,13^. Light can be naturally emitted by the sun, undergo spectrum transformation, or even be redirected. The primary energy source for traditional crop cultivation is sunlight, and the light that plants intercept in a natural setting fluctuates and is complex^14^. During cloudy days and especially in winters plants receive much less light than that required for its normal growth and development. Artificial grow lights can be used as supplemental light when natural sunshine is inadequate, and grow lights are the only source of light for vertical indoor farms with artificial lights^15,16^. Artificial lighting generates radiation that serves as both an energy source for photosynthesis and a biological signal that can modify plant morphology and quality. For effective, year-round indoor production of high-yield crops, it is important to maximize plant growth under artificial lights while simultaneously reducing expenses^17–19^. LEDs have the highest photosynthetically active radiation (PAR) efficiency of all the artificial light sources. They also allow for the optimal level of irradiance to be achieved in terms of biomass output and overall energy costs^20^. Previous studies^21,22^ have shown that plants grown under a combination of red and blue LEDs look reddish, which makes it hard to identify the signs of disease and growth problems. There were no significant changes in all the physiological and yield characteristics of lettuce plants between white LEDs and cool white fluorescent bulbs. However, white LEDs consumed less electricity, indicating that white LEDs might effectively replace classic fluorescent bulbs^23,24^.

Adequate amount of light can encourage photosynthetic activity and increase biomass production, but too much light will lead to phenomenon of photoprotection and photodamage in plants^25^. Daily light integral (DLI) is the total amount of photosynthetically active radiation (400–700 nm) delivered to plants in a 24-h period. It is a function of photoperiod and light intensity (PPFD) and indicates the amount of light needed for plant growth and development. The DLI is a crucial environmental factor for plants and demonstrates a stronger association with biomass accumulation, plant growth^26–29^, nutritional quality^30^, and daily irrigation amount^31^. However, increasing DLI deteriorated visual appearance and reduced the quantum photosynthetic yield of PSII^32^, and light use efficiency^30^. There were no significant increases in the total fresh weight and total phenolic content of sweet basil when the DLI was increased above 16.5 mol m^–2^ day^–1^^12^. Gent^33^, studied the effect of daily sunlight integral on the concentration of secondary metabolites in hydroponically grown butterhead lettuce. The study suggested that lettuce should be harvested in the afternoon after growth under high light to get maximum nutritional quality. Runkle^34^ suggested a minimum DLI of 12–14 mol m^–2^ day^–1^ for greenhouse lettuce production. Cui et al. ^29^ recommended a DLI of 6.35 mol m^–2^ day^–1^ for cucumber plug seedlings that could be achieved by using PPFD of 110–125 µmol m^−2^ s^−1^ and a photoperiod of 14–16 h. For indoor cultivation of red-leaf lettuce plants, a minimum DLI of 6.5 to 9.7 was suggested^35^. DLI requirement for lettuce ranges between 12 and 16 mol m^–2^ day^–1^, however their value varies among species and cultivars^17,22,26,36^.

Increasing PPFD has a positive effect on the rate of photosynthesis of plants up to a certain intensity at which the maximum rate of photosynthesis occurs. Further increase in PPFD results in decreased light use efficiency and plant growth^37^. This is because photoprotective activities cause absorbed light energy to be converted into heat rather than allowing it to be utilized for electron transport in the light reactions that occur during photosynthesis^38^. Many researchers have demonstrated that PPFD in the range of 150–300 µmol m^−2^ s^−1^ is optimal for cultivation of lettuce under artificial lights^26,39–42^ or even above 300 µmol m^−2^ s^−1^^43–45^. Because of the higher energy requirement, light intensities greater than 300 are generally not recommended^46^. While adding the extra photons by increasing PPFD has diminishing marginal gains, increasing photoperiod is another strategy to augment DLI and gross photosynthesis^17,47,48^.

Photoperiod is an important environmental factor regulating plant growth and development. Many plants species sense daylength to determine when to transition from vegetative growth to reproductive development. Photoperiod can influence the quantity of light plants experience in a day, the entrainment of their circadian rhythm, and the crucial dark period necessary for flowering in photoperiodically sensitive plants^49,50^. Many studies have demonstrated that plants grown under the same DLI and increasing photoperiod at low PPFD resulted in more photosynthetic activity and biomass production^38,51,52^. Additionally, Arabidopsis^53^ (Lepisto et al., 2009) and lettuce^54^ were proven to have an increase in their biomass output when exposed to continuous light. continuous lighting also increased dry and fresh shoot biomass and plant growth of lettuce (cv. Yidali) under red and blue LEDs^14^. Similar results were obtained for Nasturtium (*Tropaeolum majus* L.) plants^55^. However, in another study^17^ found that extending photoperiod from 16 to 24 h day^−1^ at DLI of 10.4 mol m^−2^ day^−1^ did not increase the dry weight of lettuce (cv. Rouxai) significantly. Additionally, CL had a detrimental impact on the rate of photosynthesis in leaves as well as the total dry weight of tomato plants^56^. It was also reported that CL has a negative impact on the fresh weight and dry weight of common ice plant^48^. Hence, the effect of photoperiod varies according to the type of crop as well as their cultivars (Supplementary Table 1).

It is important to emphasize that the plant density used in this study was in line with commercial cultivation of lettuce. This is because high-density plant spacing can result in light competition, which is not accounted for in studies that use a single plant per pot design. Therefore, it is crucial to consider high-density plant spacing to accurately evaluate the impact of light competition on plant growth and development. Both the amount of light a crop captures and how effectively it uses that light to produce biomass must be taken into account in order to accurately evaluate crop growth and productivity. One of the major disadvantages of vertical farming with artificial lights is higher electricity costs. The number of hours of light provided to plants is directly related to the cost of electricity. Hence, more research needs to be done on DLI strategies offered by sole-source white LEDs, taking energy consumption into consideration. Our study adds to the existing literature on DLI and lettuce production by assessing the impact of different DLI regimes on not only growth and yield but also nutritional quality and resource use efficiency of lettuce grown in greenhouse-based VHS, providing a more comprehensive understanding of DLI’s impact on lettuce production. Furthermore, third study compares lettuce growth under different DLI regimes provided by artificial LED lights to that of natural sunlight inside a greenhouse, allowing us to identify the optimal DLI regime for lettuce production and provide insights into the relative advantages of artificial lights in comparison to natural sunlight, which was not done before. To our knowledge, no study has examined the response of iceberg lettuce to varying DLIs using sole-source white LEDs. Very few researchers have studied the impact of DLI by varying photoperiod and keeping the PPFD constant. Therefore, the aim of our study was to study the effect of photoperiod/DLI on crop growth, photosynthesis, and quality parameters and to deter mine the optimal photoperiod/DLI for iceberg lettuce in order to optimize plant growth while taking resource use efficiency into account. We hypothesized that a higher DLI would lead to higher biomass accumulation and nutritional quality. We tested the hypotheses with three DLI treatments obtained by varying the photoperiod.

## Results

### Fresh weight

**Figure 1:**
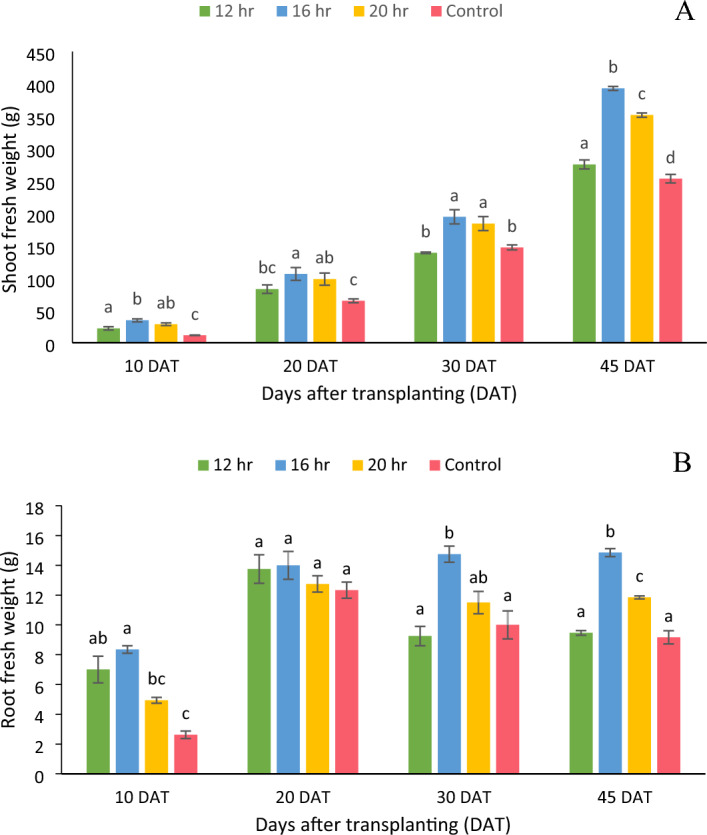
(**A**) Fresh weight of shoot and (**B**) fresh weight of root of an iceberg lettuce grown under four photoperiods (12 h, 16 h, 20 h and control) at various sampling intervals. Bars represent means ± standard error. Means followed by different letters indicate statistically significant variation at p < 0.05, according to Tukey’s HSD test.

The shoot and root fresh weight of lettuce grown in vertical hydroponic system with artificial lights were significantly affected (p < 0.05) by DLI at all sampling stages except the root FW at 20 DAT (Fig. 1). Shoot fresh weight of lettuce increased significantly with increase in DLI from 8.64 to 11.5 mol m^−2^ day^−1^, thereafter decreased significantly at DLI of 14.4 mol m^−2^ day^−1^, at the time of harvesting. Up to 30 DAT, there was no statistically significant difference (p < 0.05) in shoot FW between 11.5 and 14.4 mol m^−2^ day^−1^ treatments. Increasing the DLI from 8.64 to 11.5 mol m^−2^ day^−1^ increased shoot and root FW by 42% and 57%, respectively. However, augmenting the DLI from 11.5 to 14.4 mol m^−2^ day^−1^ decreased the shoot and root FW by 10% and 21%, respectively.

### Dry weight

**Figure 2:**
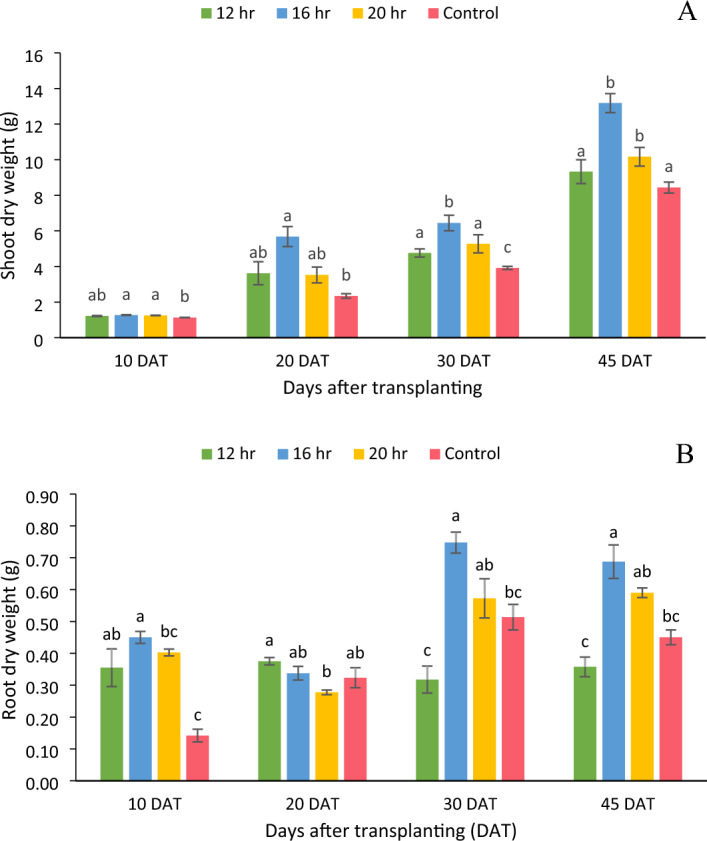
(**A**) Dry weight of shoot and (**B**) dry weight of root of an iceberg lettuce grown under four photoperiods (12 h, 16 h, 20 h, and control) at various sampling intervals. Bars represent means ± standard error. Means followed by different letters indicate statistically significant variation at p < 0.05, according to Tukey’s HSD test.

The shoot and root DW of lettuce were affected significantly (p < 0.05) at all the sampling stages by DLI treatments (Fig. 2). A similar trend was observed in DW as in the case of FW except that there was no significant difference in shoot and root DW between the control and 8.64, 11.5 and 14.4 mol m^−2^ day^−1^ DLI at the harvesting stage. Only at 30 DAT, the significant difference was observed between control and 8.64, 11.5 and 14.4 mol m^−2^ day^−1^ DLI. A 41% increase in shoot DW was observed by increasing DLI from 8.64 to 11.5 mol m^−2^ day^−1^, but a further increase in DLI to 14.4 mol m^−2^ day^−1^ resulted in a 23% decrease in shoot DW. The 8.64 mol m^−2^ day^−1^ DLI had 11% more shoot dry weight than the control treatment but the difference was not statistically significant.

### Leaf area

**Figure 3:**
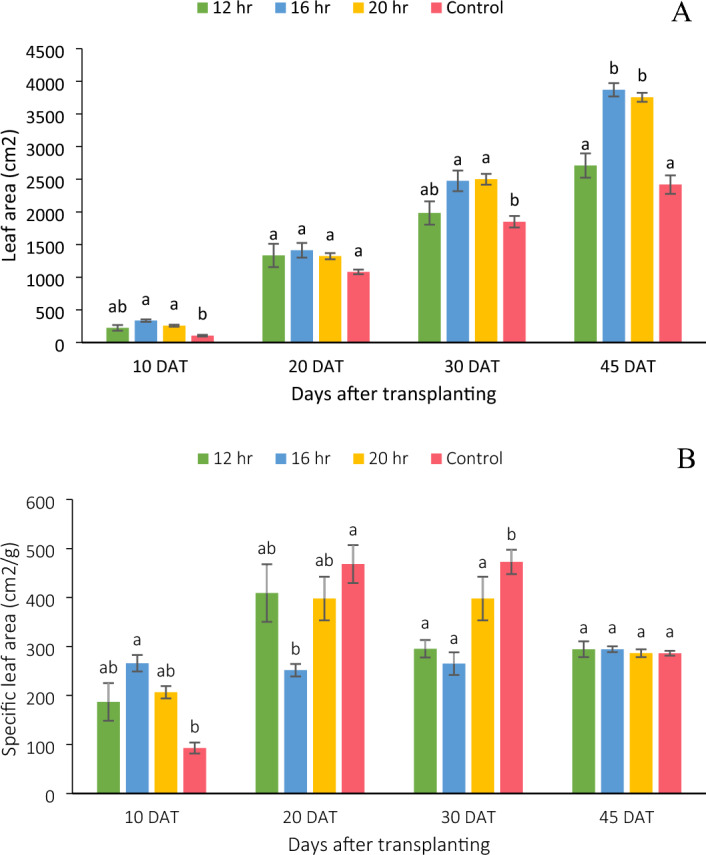
(**A**) Leaf area of lettuce and (**B**) specific leaf area of lettuce at different sampling stages. Bars represent means ± standard error. Means followed by different letters indicate statistically significant variation at p < 0.05, according to Tukey’s HSD test.

Leaf area of an iceberg lettuce grown in indoor hydroponic system showed a distinct response to DLI/photoperiod. Leaf area increased by 43% with increase in DLI from 8.64 to 11.5 mol m^−2^ day^−1^ (Fig. 3A), further increment in DLI to 14.4 mol m^−2^ day^−1^, slightly decreased the leaf area (3%). However, the decrease in leaf area at 14.4 mol m^−2^ day^−1^ DLI was not statistically significant. No significant difference in all the treatments were found at 20 DAT. Also, the 8.64 mol m^−2^ day^−1^ DLI had 12% more leaf area compared to control treatment but the difference was not statistically significant.

### Specific leaf area

DLI/Photoperiod did not significantly (p < 0.05) affect the specific leaf area of lettuce at the time of harvest. There was no consistent trend over the course of time between the photoperiod and specific leaf area (Fig. 3B). The specific leaf area for 8.64, 11.5 and 14.4 mol m^−2^ day^−1^ DLI treatments were not substantially different at all the sampling stages.

### Number of leaves

**Figure 4:**
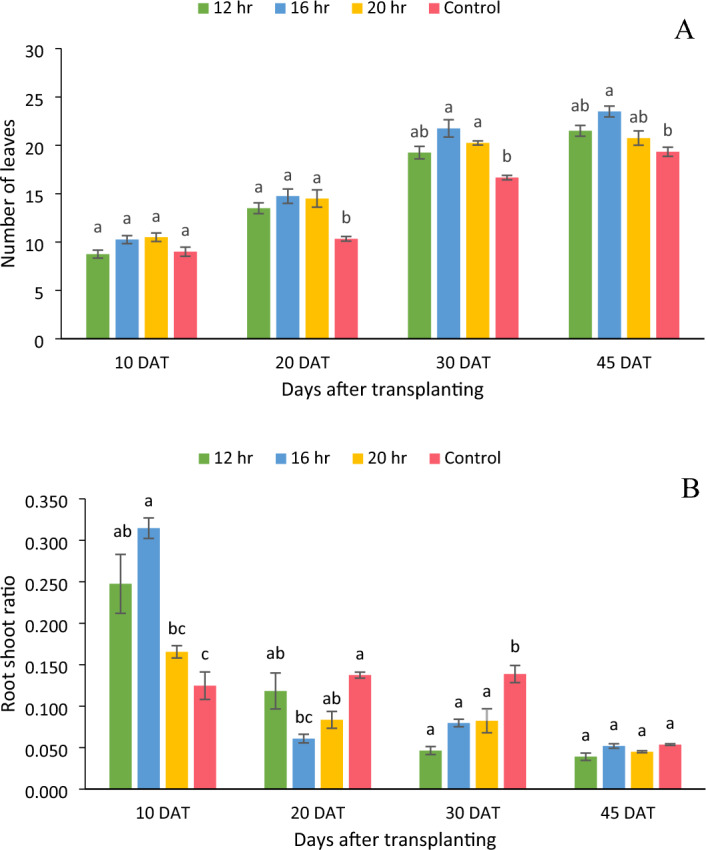
(**A**) Number of leaves per plant and (**B**) Root shoot ratio of per plant at different sampling stages. Bars represent means ± standard error. A significant (p < 0.05) variation in means is denoted by different letters according to Tukey’s HSD test.

The number of leaves were significantly (p < 0.05) higher in the case of 11.5 mol m^−2^ day^−1^ DLI treatment compared to control treatment at the time of harvesting (Fig. 4A). The 11.5 mol m^−2^ day^−1^ DLI had the highest average number of leaves (24), followed by 8.64 (22), 14.4 (21), and control (19). There was no significant difference between all DLIs/ photoperiods at 10 DAT. It was also found that the DLI provided by artificial lights (8.64, 11.5, 14.4) have not substantially affected the number of leaves.

### Root shoot ratio

A decreasing trend was observed in root shoot ratio at each successive sampling stage (Fig. 4B). There was no significant difference between 8.64, 11.5, and 14.4 mol m^−2^ day^−1^ DLI treatments at all the sampling stages except at 10 DAT. The 8.64 mol m^−2^ day^−1^ DLI had the lowest root shoot ratio (0.039) followed by14.4 mol m^−2^ day^−1^ (0.045) and 11.5 mol m^−2^ day^−1^ (0.052) whereas the control treatment had the highest root shoot ratio at the time of harvesting but their differences were not significant.

### Leaf gas exchange analysis

**Figure 5:**
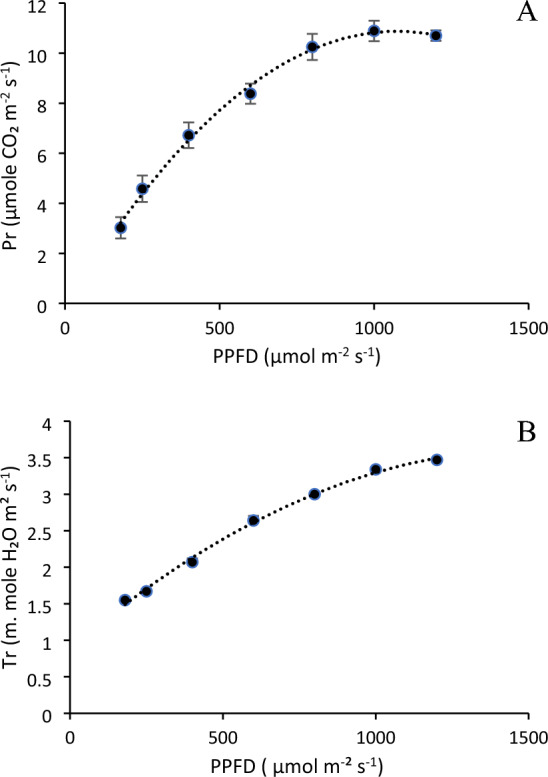
Response of (**A**) photosynthetic rate and (**B**) transpiration rate of lettuce leaves to increasing light intensity from white LED lights. Error bars represents standard error of the mean.

The light response curve showed a good correlation between the photosynthetic rate and PPFD. Photosynthetic rate increased continuously from 3.02 µmol CO_2_ m^−2^ s^−1^ at 200 µmol m^−2^ s^−1^ to 10.89 µmole CO_2_ m^−2^ s^−1^ at 1000 µmol m^−2^ s^−1^, where it reached a light saturation point (Fig. 5A). Further increase in PPFD to 1200 µmol m^−2^ s^−1^ decreased the photosynthetic rate (by 2%) to 10.7 µmole CO_2_ m^−2^ s^−1^. The response curve for transpiration rate (Fig. 5B) showed a continuous increasing trend with increasing PPFD unlike Pr, where it was decreased after increasing PPFD from 1000 µmol m^−2^ s^−1^ to 1200 µmol m^−2^ s^−1^. Augmenting PPFD from 400 to 600 µmol m^−2^ s^−1^, increased the transpiration rate by 28%. However, further increase in PPFD to 800 and to 1000 µmol m^−2^ s^−1^ resulted in 14% and 11% respectively.

For analyzing leaf gas exchange parameters, the control treatment which consisted of a three-layer VHS without artificial lights, was divided into top, middle, and bottom layer. We have studied the response of leaf gas exchange parameters per layer in control treatment consist of a three-layer VHS without artificial lights which was then compared with the VHS with artificial lights. We observed, for control condition that the photosynthetic activity of lettuce decreased substantially from top layer to bottom layer by 70% (Table 1). Increasing DLI from 8.64 to 11.5 mol m^−2^ day^−1^, increased the photosynthetic activity by 22%. However, no significant difference was observed between 11.5 and 14.4 mol m^−2^ day^−1^. Water use efficiency (Pn/E) was also found to be decrease from top layer to bottom in control treatment. The highest WUE was observed under a DLI treatment of 11.5 mol m^−2^ day^−1^ corresponding to a photoperiod of 16 h.

**Table 1:** Photosynthetic rate, stomatal conductance, Intercellular CO_2_ concentration, Transpiration rate, water use efficiency (WUE) and intercellular to ambient CO2 concentration (Ci/Ca) of iceberg lettuce expressed as mean ± standard error, subjected to different DLI levels.

Treatments	Photosynthetic rate (Pn)	Stomatal Conductance	Intercellular CO_2_ concentration	Transpiration rate (E)	WUE (Pn/E)	Ci/Ca
CT	8.06 ± 0.02 a	0.16 ± 0.001 b	357.64 ± 0.59 b	1.57 ± 0.001 ab	5.14 ± 0.009 c	0.90 ± 0.001 a
CM	6.67 ± 0.07 b	0.14 ± 0.001 b	404.95 ± 0.35 a	1.45 ± 0.008 a	4.59 ± 0.005 bc	0.81 ± 0.002 b
CL	4.74 ± 0.03 c	0.20 ± 0.004 a	361.55 ± 0.94 b	1.38 ± 0.002 a	3.44 ± 0.015 a	0.79 ± 0.001 b
12 h/8.64 DLI	7.06 ± 0.23 db	0.09 ± 0.001 cd	398.84 ± 0.98 a	1.46 ± 0.004 a	4.84 ± 0.017 bc	0.88 ± 0 .005 a
16 h/11.5 DLI	8.60 ± 0.21 a	0.13 ± 0.004 bd	445.20 ± 0.32 c	1.48 ± 0.009 a	6.03 ± 0.042 d	0.95 ± 0.003 c
20 h/14.4 DLI	7.90 ± 0.36 a	0.12 ± 0.002 d	462.80 ± 0.88 d	1.89 ± 0.002 b	4.22 ± 0.021 b	0.94 ± 0.002 c

Means followed by similar letter in a same column represents no significant difference (*CT* control top layer, *CM* control middle layer, *CB* control bottom layer).

#### Qualitative parameters

All the qualitative parameters of lettuce were varied widely in response to changing photoperiod. Total phenolic content of 11.5 mol m^−2^ day^−1^ DLI (15.27 ± 0.49 mg GAE/100 g of FW) was significantly (p < 0.05) higher compared to 14.4 and control (Table 2). Total phenols were 2.5% higher in the 11.5 mol m^−2^ day^−1^ DLI than in the 8.64 mol m^−2^ day^−1^ DLI, which was not statistically significant. Additionally, total phenols in all the photoperiod treatments provided by artificial lights were substantially higher than that of control. The photoperiod had no effect on the antioxidant capacity of lettuce grown under artificial lights. However, it was significantly (p < 0.05) higher compared to control. The highest antioxidant capacity (8.48 ± 0.17 µmol Trolox/g FW) was observed under 11.5 mol m^−2^ day^−1^ DLI followed by 14.4 mol m^−2^ day^−1^ (8.05 ± 0.14 µmol Trolox/g FW) DLI. The antioxidant capacity of 11.5 mol m^−2^ day^−1^ DLI was 29.70% higher than the control. Vitamin C content of iceberg lettuce increased with increasing DLI from 11.5 to14.4 mol m^−2^ day^−1^. However, there was no significant difference between8.64 and 11.5 mol m^−2^ day^−1^ DLI. Vitamin C content was found to be highest under14.4 (14.38 mg/100 g) period whereas the lowest was observed in case of control treatment (12.10 mg/100 g)0.14.4 mol m^−2^ day^−1^ DLI had 19% more vitamin C content than the control. The physiological parameters like leaf length, root length and plant height were also measured at time of harvest. Leaf length was not affected photoperiod of artificial LED lights but it was significantly higher compared to control. Root length had no significant difference among all treatments. Plant height was substantially higher for 11.5 mol m^−2^ day^−1^ DLIs compared to 14.4 mol m^−2^ day^−1^ and control treatments. However, there was no significant difference in plant height between 8.64 and 11.5 mol m^−2^ day^−1^ treatments.

**Table 2:** Qualitative and physiological characteristics of an iceberg lettuce affected by different photoperiods at the time of harvest.

Treatments	Total phenols	Antioxidant capacity	Vitamin C	Leaf length	Root length	Plant height
	(mg GAE /100 g)	(µmol Trolox/g FW)	(mg/100 g)	(cm)	(cm)	(cm)
12 h/8.64 DLI	14.90 ± 0.60 a	7.83 ± 0.16 a	13.13 ± 0.19 a	20.38 ± 0.89 a	39.62 ± 1.32 a	58.25 ± 0.49 ab
16 h/11.5 DLI	15.27 ± 0.49 a	8.48 ± 0.17 a	13.28 ± 0.25 a	17.50 ± 0.72 a	44.63 ± 0.66 a	65.50 ± 1.5 a
20 h/14.4 DLI	11.93 ± 0.37 b	8.05 ± 0.14 a	14.38 ± 0.23 c	17.00 ± 0.42 a	37.00 ± 2.70 a	55.25 ± 2.22 b
Control	8.23 ± 0.45 c	6.63 ± 0.15 b	12.10 ± 0.09 b	13.42 ± 2.61 b	29.43 ± 6.93 a	43.43 ± 10.65 b

Values are represented as means ± standard error.

Means followed by different letters denotes significant difference at p < 0.05 according to Tukey’s HSD test.

### Resource use efficiency

#### Energy use efficiency

**Figure 6:**
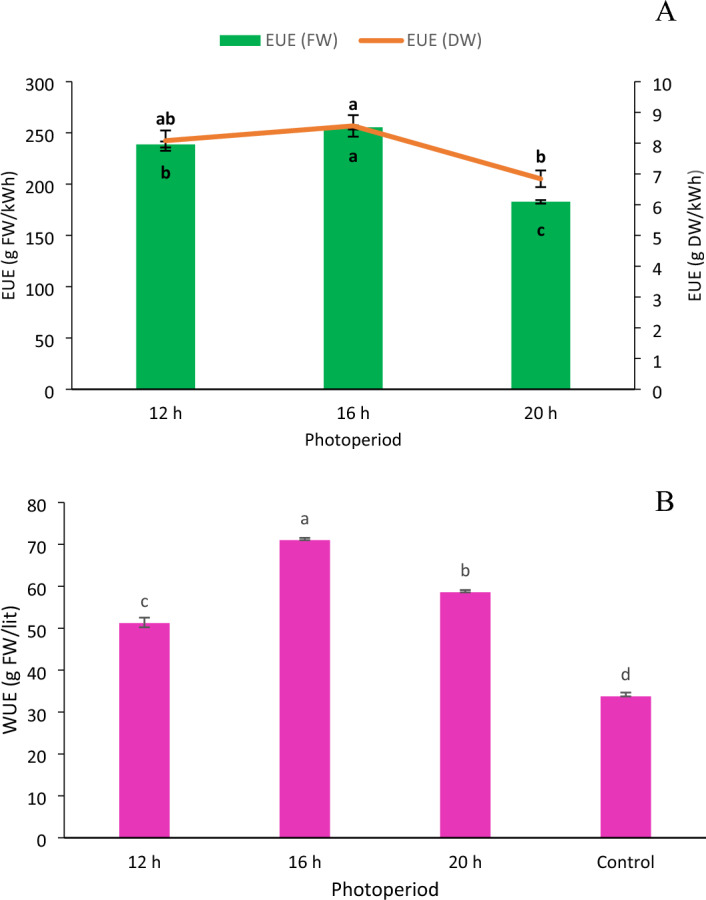
(**A**) Energy use efficiency and (**B**) water use efficiency of an iceberg lettuce grown under four photoperiods. Bars represent means ± standard error. A significant (p < 0.05) variation in means is denoted by different letters according to Tukey’s HSD test.

Electricity was necessary to power the submersible pump, aerator pump and LEDs. The electric energy consumed by LEDs was considered to compute electric EUE. The 8.64 mol m^−2^ day^−1^ DLI required the least amount of energy (41.55 kWh) compared to the other two DLIs. Varying DLI had significant impact on EUE of lettuce (Fig. 6A). Increasing the DLI from 8.64 to 11.5 mol m^−2^ day^−1^ boosted EUE by 7%. The EUE reduced by 28% as the DLI further increased to 14.4 mol m^−2^ day^−1^. The 11.5 mol m^−2^ day^−1^ DLI produced the highest EUE, whereas the14.4 mol m^−2^ day^−1^ DLI produced the lowest.

#### Water use efficiency

The water use efficiency of lettuce was significantly (p < 0.05) affected by different photoperiods (Fig. 6B). The total water consumption was highest (262.82 lit) in control treatment compared to all other treatments. In case of artificial light treatments, the 8.64 mol m^−2^ day^−1^DLI used the least amount of water (193.58 lit). The 11.5 mol m^−2^ day^−1^ DLI had the maximum water use efficiency (100.34 g FW L^−1^ H_2_O) followed by14.4 mol m^−2^ day^−1^ (82.76 g FW L^−1^ H_2_O), whereas control treatment had the lowest (72.39 g FW L^−1^ H_2_O). WUE increased by 38.6% when the DLI was extended from 8.64 to 11.5 mol m^−2^ day^−1^. A further increase in DLIto14.4 mol m^−2^ day^−1^ reduced WUE by 17.5%. With artificial light treatments, 5.64 lit of water was consumed per plant, compared to 7.3 lit of water under sunlight treatment inside the greenhouse.

## Discussion

Several studies have examined the effect of variation in DLI due to changes in PPFD on a variety of indoor-grown plants, including lettuce^26,28,36,61^. However, few studies, primarily focusing on lettuce, have examined the effects of modifying photoperiod in indoor cultivation under LED lighting^25,42,62,63^. In the present study, the effect of DLI obtained by varying photoperiod (12 h, 16, and 20 h) with constant PPFD of 200 µmol m^−2^ s^−1^ on iceberg lettuce grown in a vertical hydroponic system under white LED lights was investigated. It was observed that the growth, nutritional quality and resource use efficiency of iceberg lettuce was optimized at 16 h photoperiod DLI = 11.52 mol m^−2^ day^−1^).

### Effect of photoperiod/DLI on growth and morphological parameters of lettuce grown in vertical hydroponic system

The yield of crop has a direct relationship with DLI^64,65^. Increase in DLI increased the growth and morphological characteristics of an iceberg lettuce. Fresh and dry weight of leaf and root was boosted significantly (p < 0.05) with increase in DLI from 8.64 to 11.5 mol m^−2^ day^−1^. Our results were in line with previous studies on the same crop with different cultivars and growing conditions indicating increase in fresh and dry weight with increasing DLI^22,26,28,32^. Yan et al.^22^ found that the greatest yield of mature leaf lettuce was observed at for the combination of 200 µmol m^−2^ day^−1^ and a photoperiod of 16 h ((DLI = 11.5 mol m^−2^ day^−1^) using four different LED lights provided at seedling stage. In our study, we found the similar trend at harvesting stage where the LED light treatment was provided after transplanting. The CO_2_ concentration maintained by Yan et al.^22^ was almost double (800 ± 50 μmol·mol^−1^) than our study. They tested the effects of two different photoperiods (14 and 16 h) and two different levels of PPFD (200 and 250 µmol m^−2^ day^−1^) while exposing the lettuce seedlings to four different light quality conditions.

Dry biomass of lettuce increased substantially as DLI increased from 8.64 to 11.5 mol m^−2^ day^−1^. Increase in dry weight was associated with increased leaf area of lettuce. However, further increment in DLI to14.4 mol m^−2^ day^−1^ decreased the fresh weight substantially demonstrating that an excessive amount of DLI had a negative impact on the accumulation of carbohydrates and even caused a reduction in the accumulation of dry weight^30,35,66^. There was no significant difference in dry weight of shoot and root between 11.5 and 20 h photoperiod. These results were in line with previous studies^26,64^. Increasing DLI from 11.5 to 14.4 mol m^−2^ day^−1^ decreased fresh weight and dry weight of leaves by 10% and 23% respectively. This may be explained by Photoinhibition which is defined by Xu and Shen^65^, as a light-induced decrease in photosynthetic efficiency that occurs when plants receive more light energy than they require for photosynthesis. Furthermore, the closure of stomatal pores and the subsequent reduction in gas exchange may account for the inferior performance of 20 h photoperiod^67^ (DLI = 14.4 mol m^−2^ day^−1^). It was previously demonstrated that the fresh weight of green and red lettuce did not vary substantially in response to DLI levels ranging from 13 to 26 mol m^−2^ day^−1^^25^. Leaf area of lettuce was increased by 43% with increase in DLI from 8.64 to 11.5 mol m^−2^ day^−1^ under LED lights. Similar results were obtained by Refs.^62,68,69^. However, Palmer and van lersel^63^ found that different photoperiods (10, 12, 14, 16, 18, and 20 h) at constant DLI of 16 mol m^−2^ day^−1^ had no influence on leaf area of lettuce and mizuna. Specific leaf area of lettuce at harvest was not substantially influenced by photoperiod. Similar results were obtained by (Weaver and van lersel, 2020) for ‘Little Gem’ lettuce at constant DLI of 17 mol m^−2^ day^−1^.

### Effect of photoperiod/DLI on nutritional quality of lettuce

The total phenolic content of lettuce increased with increase in DLI from 8.64 to 11.5 mol m^−2^ day^−1^ and then decreased at 14.4 mol m^−2^ day^−1^. The decrease in phenolic content at DLI of 14.4 mol m^−2^ day^−1^ might be due to the achievement of light saturation point. This result was in accordance with Cho et al.^69^, where they have used external electrode fluorescent lamps to study the impact of various combinations of photoperiod and PPFD on leaf lettuce. Carvalho et al.^70^, observed that the total phenolic content in leaves of sweet potato was higher at 16 h photoperiod compared to 8 h photoperiod with PPFD of 150 µmol m^−2^ day^−1^. Antioxidants found in nutrient-dense plant foods, such as fruits and vegetables, are crucial for lowering the risk of oxidative stress-related chronic diseases^71,72^. Ali et al.^73^ observed that the levels of antioxidant activity in swiss chard, red beet, green amaranth, red amaranth and red spinach were found to be higher at a photoperiod of 12 h. Antioxidant capacity of lettuce was found to be highest under a combination of 12 h photoperiod and half strength of nutrient solution^74^. Cho et al.^69^, demonstrated that the antioxidant capacity of green leaf lettuce increased up to 20 h and then decreased at 24 h. In our study, we observed that the antioxidant capacity was increased up to A DLI of 11.5 mol m^−2^ day^−1^ and then decreased at 14.4 mol m^−2^ day^−1^. Antioxidant activity has been demonstrated by about 70% of the phenolic substances found in lettuce^75^, suggesting that the trends in total phenolic content and antioxidant level in this study may be similar to each other. Vitamin C content of iceberg lettuce increased with increasing DLI. Several other researchers reported similar trend^26,76^.

### Effect of photoperiod/DLI on resource use efficiency of lettuce grown in vertical hydroponic system

The higher energy demand is one of the barriers in the widespread adoption of indoor vertical farming with artificial lights. Hence, increasing resource use efficiency helps to reduce costs and improve the system’s overall sustainability. It can also assist in ensuring that the plants receive a sufficient amount of resources, which can improve their growth and yield. By optimizing resource use efficiency, the productivity as well as profitability of indoor hydroponic system can be increased while, also reducing their environmental impact. In the present study, WUE of lettuce was increased with increase in DLI up to 11.5 mol m^−2^ day^−1^ and then decreased thereafter. The highest WUE of 71 g FW/lit was observed at DLI of 11.52 mol m^−2^ day^−1^. Our findings were in line with Pennisi et al.^77^ but with higher DLI, where they observed that the WUE of butterhead lettuce (cv. Rebelina) was increased successively by increasing PPFD from 100 µmol m^−2^ day^−1^ (DLI = 5.8 mol m^−2^ day^−1^) to 250 µmol m^−2^ day^−1^ (DLI = 14.4 mol m^−2^ day^−1^). Increasing PPFD more than 250 µmol m^−2^ s^−1^, resulted in a slight decrease in WUE. They observed the highest WUE of 60 g FW/lit, which is less than our findings. Further, Pennisi et al.^42^, observed that changes in photoperiod had no impact on WUE of butterhead lettuce (cv. Rebelina). WUE observed in the present study is far better than the WUE of lettuce reported in traditional open field and greenhouse condition. Barbosa et al.^78^ reported WUE of lettuce in open field and greenhouse hydroponic system as 4 g FW/lit and 20 g FW/lit respectively.

In the present study, energy requirement increased with increase in DLI. The maximum EUE of 206.31 g FW/kWh was observed under a DLI of 11.5 mol m^−2^ day^−1^. Zhang et al.^26^ demonstrated that the EUE of lettuce was increased from 15.9 to 40.6 g FW/kWh by shifting from fluorescent lights to LED lights. Yan et al.^79^ studied the effect of combination of various LEDs on EUE of green and purple leaf lettuce. They found that the maximum EUE of 80 g FW/kWh was observed under white LED lights. Pennisi et al.^42^ revealed that the EUE of butterhead lettuce decreased with increase in photoperiod from 16 to 24 h at PPFD of 250 µmol m^−2^ s^−1^ under blue and red LEDs. The maximum EUE of 138 g FW/kWh was observed under 16 h photoperiod corresponding to a DLI of 14.4 mol m^−2^ day^−1^. Improvements in the intrinsic characteristics of the light source, particularly the light's energy usage and photosynthetic photon efficacy, are primarily responsible for the significant increases in EUE observed in the present study^80^. The photosynthetic photon efficacy of LED lights used in our study was 2.2 µmol/J which was more than 1.52 µmol/J used by Pennisi et al.^42^.

## Conclusion

It is essential to comprehend the response of plants to varying lighting conditions in order to determine the optimal light recipe. To ensure optimal plant growth under artificial lighting, the relationship between PPFD, photoperiod, DLI, and resource use efficiency should be considered when developing an optimal light control algorithm. Growers often wants to know the minimum PPFD and duration for optimal growth of crops to reduce the cost of cultivation in addition to resource use efficiency. This is the first study to demonstrate the response of an iceberg lettuce grown in vertical hydroponic system to various DLI obtained by altering photoperiod. Our study demonstrated that altering DLI by modifying photoperiod had a substantial effect on the growth and development of iceberg lettuce. We observed that increasing DLI by changing photoperiod from 8.64 (DLI = 8.64 mol m^−2^ day^−1^) to 16 h (DLI = 14.4 mol m^−2^ day^−1^) resulted in significant increase in carbohydrate accumulation, leaf area, and resource use efficiency of an iceberg lettuce, while 20 h photoperiod (DLI = 14.4 mol m^−2^ day^−1^) resulted in marginal decrease in physiological and morphological parameters and a significant decrease in resource use efficiency. Thus, from our study, we can conclude that the optimal growth, nutritional quality, and resource use efficiency of an iceberg lettuce was achieved at a photoperiod of 16 h corresponding to a DLI of 11.5 mol m^−2^ day^−1^ and a PPFD of 200 µmol m^−2^ s^−1^ under white LED lights. The study has demonstrated a practical light management strategy for growing iceberg lettuce under indoor vertical hydroponic system using minimal resources. Our findings suggest that determining an optimal DLI regime for lettuce production, farmers could potentially reduce lighting energy costs while also improving the nutritional quality of crop, which could have far-reaching implications for sustainable agriculture.

The present study revealed that the nutritional quality of lettuce improved up to threshold DLI, after which it decreased. Whereas in case of antioxidant content no significant change was observed under all DLI treatments. The study demonstrates that by manipulating the lighting conditions within the optimal range, growers can achieve higher resource use efficiency in VHS. Increasing the DLI within a specific range resulted in significant improvements in carbohydrate accumulation, leaf area, and resource use efficiency of the lettuce. This study has demonstrated a cost-effective light management strategy for indoor cultivation of iceberg lettuce in greenhouse based indoor VHS.

## Materials and methods

### Plant material and growing conditions

The present experiment was carried out in a climate-controlled greenhouse located at the Centre for Protected Cultivation Technology, ICAR-Indian Agricultural Research Institute, New Delhi, India. The seeds of iceberg lettuce (*Lactuca sativa* cv. Glendana, Enza Zaden, North Holland, Netherlands) were sown in a plastic plug tray (45 × 70 cm) filled with a mixture of coco-peat, perlite, and vermiculite in a ratio of 3:1:1 (w/w), respectively. For three days, the plug trays were covered with translucent plastic domes to maintain a high level of humidity. After germination, plants were trimmed to one plant per cell. The trays were kept in a nursery greenhouse for about 3 weeks. The trays were watered as per the requirement. ¼ strength nutrient solution was provided three days after sowing, cotyledon stage and 1–2 true leaves stage. The seedlings after 3 weeks were transplanted to an indigenous NFT (Nutrient Film Technique) type vertical hydroponic system (VHS) (1220 mm × 610 mm × 1520 mm) having three layers. Each layer can accommodate 36 plants. The distance between each layer was kept at 35 cm. The outer rows of plants in each plot were not included in the analysis because they were considered border plants. To maintain the original planting density, plants were moved after each destructive harvest. The system was kept in a bamboo structure enclosed by black and white shade net inside greenhouse to block any sunlight falling on the structure. The lettuce plants were harvested 45 days after transplanting (DAT). The entire structure was enclosed using a rectangular shade net to block sunlight. Additionally, a polystyrene sheet (1220 mm × 610 mm) was fixed on top of each layer to prevent the entry of light from the upper layer.

A 30-L capacity reservoir was provided per treatment to store and supply nutrient solution. The nutrient solution consisted of the following components (mg L^−1^): KH_2_PO_4,_ 200; NH_4_H_2_PO_4_, 10; KNO_3_, 630; K_2_SO_4_, 200; Ca (NO_3_)_2_·4H_2_O, 1000; MgSO_4_, 200; Fe, 40; Cu, 0.4; Zn, 3; Mn, 3; Na_2_[B_4_O_5_(OH)_4_] ·8H_2_O, 4; Na_2_MoO_4_, 0.2, respectively. The seedlings, after transplanting, were watered for two days before providing the nutrient solution. The electrical conductivity (EC) of the nutrient solution was maintained between 1.2 and 2 dS/m using water and nutrient solution. The pH of the nutrient solution was maintained at 5.5–6.5, using acidic (1 M, H_2_SO_4_) and basic (1 M, KOH) solutions. Fresh nutrient solution was added to the reservoir when the water level reached near the pump at the bottom. The nutrient solution was renewed every 2 weeks after transplantation. The mean temperature (mean ± SD) and relative humidity inside the chamber were 20.1 ± 3.3 °C and 69.61 ± 7.6%, respectively (with ambient CO_2_ concentration). The methods employed in this study were conducted in strict adherence to the relevant guidelines and regulations set forth by relevant national and international guidelines, ensuring the scientific integrity and ethical soundness of the research presented in this report.

### Light treatments

**Figure 7:**
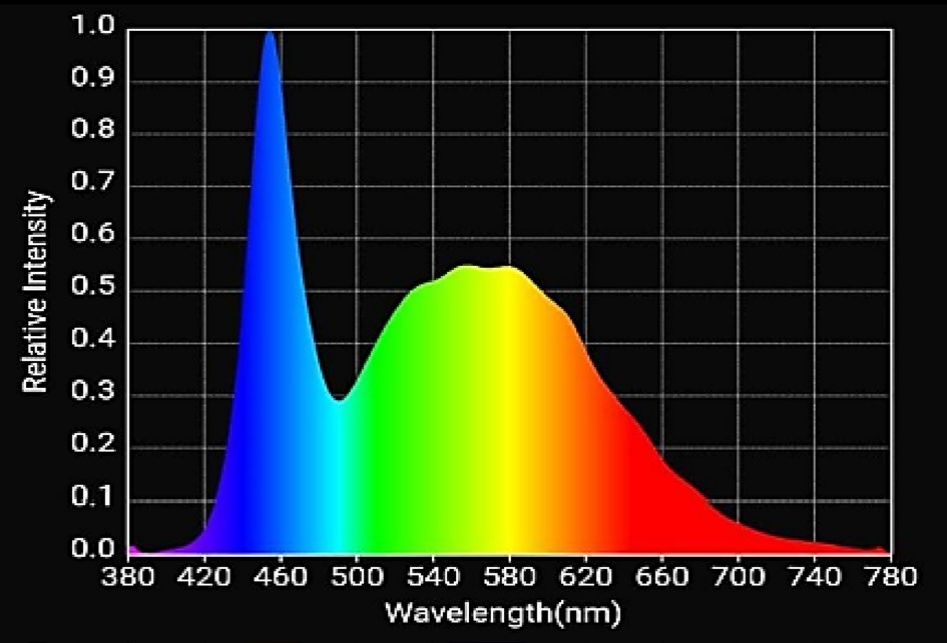
Details of light spectra of the LED used in the experiment (source: https://www.nexsel.tech/).

**Figure 8:**
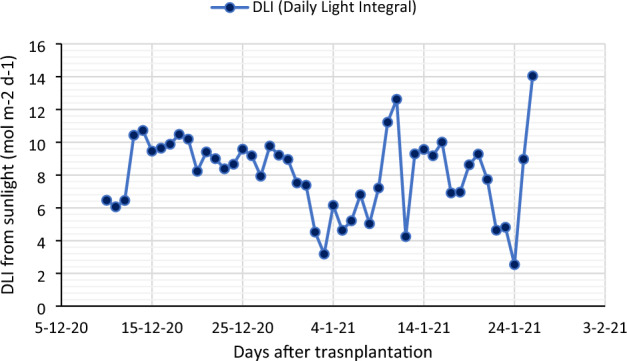
Variation of DLI from sunlight inside greenhouse.

The artificial light was provided by white LED tubes (Model-HYGL8, spectrum N × 4, 17 Watt, Nexel Tech Pvt. Ltd., India) with R:B ratio of 2.6. Details of the light spectrum are shown in Fig. 7. Four white LEDs were mounted uniformly on top of each layer. A distance of 15 cm was maintained between each LED to get uniform PPFD. A quantum sensor LI-190 (Li-Cor, Lincoln, Nebraska, USA) was used to measure the photosynthetic photon flux density (PPFD) over the plant canopy. The PPFD output of LED tubes was maintained at 200 ± 10 µmol m^−2^ s^−1^. The photoperiods of 12, 16, and 20 h were provided by connecting LED tubes to a timer with a corresponding DLI value of 8.64, 11.5 and 14.4 mol m^−2^ day^−1^, respectively (Table 3). Calculations for the Daily Light Integrals (DLI) were made by multiplying the PPFD (in µmol m^−2^ s^−1^) by the photoperiod (in seconds), and the results were given as mol m^−2^ day^−1^. The growth of plants under artificial light was compared with the plants grown in a vertical hydroponic system (NFT) under sunlight in the greenhouse condition. The daily variation of DLI (in mol m^−2^ day^−1^) received from sunlight is depicted in Fig. 8. DLI was computed by using solar radiation data from automatic weather station located inside greenhouse The solar radiation data in W m^−2^ was converted to PPFD in µmol m^−2^ s^−1^ by using a conversion factor of 2.06^57^.

**Table 3:** The DLI treatments provided by LED lights.

Photoperiod (h)	Light source	DLI (mol m^−2^ day^−1^)	PPFD (µmol m^−2^ s^−1^)
12	White LED light	8.64	200
16	White LED light	11.5	200
20	White LED light	14.4	200

### Growth measurements

#### Plant morphological parameters

The growth measurements were taken from three plants of each treatment at an interval of 10 days after transplanting. The following parameters were measured from sampled plants: Fresh and dry weight of shoot and root was measured at 10 days interval from transplanting to harvest using an electronic weighing balance. The harvested plants were dried in an oven at 60 °C for 72 h and then the measurements of dry weight of root and shoot were taken. Number of leaves per plant (length more than 2 cm) were counted and recorded. The leaf area was measured using LI-3100C leaf area meter (LI-COR Inc., Lincoln, Nebraska). The Specific Leaf Area (SLA) was determined by taking the ratio of leaf area of the plant to its dry weight.

#### Resource use efficiency

The total water use per treatment was computed separately and the water use efficiency was calculated from the ratio of plant fresh weight to the irrigation water used by plants expressed in g FW L^−1^ H_2_O. The electric energy use efficiency was calculated from the ratio of plant fresh weight at harvest to the total electricity consumption of LEDs expressed as g FW kWh^−1^ and g DW kWh^−1^.

#### Leaf gas exchange analysis

A portable photosynthesis system (LI-6400*xt*, LI‐COR Inc., Lincoln, USA) was used to record light response curves and gas exchange characteristics like photosynthetic rate (Pn, µmol CO_2_ m^−2^ s^−1^), stomal conductance (mol H_2_O m^−2^ s^−1^), transpiration rate (E, m. mole H_2_O m^−2^ s^−1^), and the ratio of intercellular to ambient CO_2_ concentration, etc. After achieving steady state and equilibrium conditions, measurements were taken on a physiologically active leaves (44 DAT) from each plant between 9 a.m. to 11.30 a.m. The average vapor pressure deficit (VPD) in the leaf chamber was 2.2 kPa. Readings were taken when sub-stomatal CO_2_ concentration (Ci) reached a stable value.

### Qualitative parameters

#### Total phenols

The total phenolic content of fresh lettuce samples was determined according to Ainsworth and Gillespie^58^. 1 g of fresh lettuce samples from each treatment were homogenized in 20 ml ethanol (80%). The crushed samples were centrifuged at 4 °C for 20 min at 10,000 rpm. An aliquot of 0.1 ml was mixed with 0.5 ml Folin–Ciocalteu reagent and left for 5 min. Following the addition of 2 mL of 20% Na_2_CO_3_, the remaining volume was brought up to 20 mL with 80% ethanol. One-centimeter cuvette was used in a spectrophotometer (Spectra Max M2, Molecular Devices, USA) to measure absorbance at 765 nm.

#### Antioxidant activity

A test for a DPPH free-radical scavenging effect was carried out according to Mensor and others^59^. A fresh sample of lettuce of 1 g was taken and crushed in 20 ml Methanol. The samples were centrifuged at 4 °C for 20 min at 10,000 rpm. A mixture of 1 mL of extract solution and 3.9 mL of DPPH was kept in the dark for 30 min. A UV–VIS Spectrophotometer (Spectra Max M2, Molecular Devices, USA) was used to record the absorbance of the reaction mixture at the 517 nm wavelength. The percentage of DPPH inhibition was computed as follows:

$${\text{Scavenging effect }}\left( {\% {\text{ DPPH inhibition}}} \right) \, = {1 } - \, \left( {{\text{A}}_{{1}} /{\text{A}}_{0} } \right)] \times {1}00,$$

where A_1_ = Sample absorbance reading at 517 nm, A_0_ = Blank absorbance reading at 517 nm, Methanol (95%) was used as a blank. The results were presented in µmol Trolox/g FW.

#### Ascorbic acid

The volumetric approach with 2, 6-dichlorophenol-indophenol dye was utilized for the determination of ascorbic acid^60^. 2 g of lettuce sample was crushed in 100 mL Metaphosphoric acid (HPO_3_) solution of 3% concentration. Following that, the sample was filtered through Whatman No. 1 filter paper. 10 mL of filtered solution was transferred to a conical flask and titrated with a dye until a pink hue appeared. The readings were noted and the results were expressed in mg per 100 g.

## Experimental design and statistical analysis

The experiment was designed with a Completely Randomized Design (CRD) with three replications. Each layer of the vertical hydroponic system contained four NFT channels. Each NFT channel was treated as a separate replication. The plants were destructively sampled at the desired intervals (10, 20, 30 and harvest) and growth parameters were recorded. Three plants were sampled from each treatment to assess growth characteristics. The data obtained was analyzed by using SPSS software (International Business Machines Corporation, Chicago, United States) to measure the significance of variance among different parameters in three photoperiods. The univariate analysis was performed using Tukey’s honest significance difference (HSD) test at p < 0.05 was used to measure significant difference in means among treatments. The ANOVA tables obtained for each parameter are presented in a supplementary data file (Supplementary information).

## Supplementary Information

Supplementary Information.

## Data Availability

The datasets used and/or analyzed during the current study are available from the corresponding author on reasonable request.
