# Supplementary material for: Determination of optimal daily light integral (DLI) for indoor cultivation of iceberg lettuce in an indigenous vertical hydroponic system
Source: Sci Rep. 2023 Jul 5;13:10923. doi: 10.1038/s41598-023-36997-2 (PMC10322904; doi:10.1038/s41598-023-36997-2)
Supplement: Supplementary file 1 — Supplementary Information. [file 41598_2023_36997_MOESM1_ESM.docx]

**Table 4a**: ANOVA table for shoot fresh weight at harvesting stage.

| **Source** | **Type III Sum of Squares** | **df** | **Mean Square** | **F** | **Sig.** |
| --- | --- | --- | --- | --- | --- |
| Corrected Model | 54843.250^a^ | 3 | 18281.083 | 237.803 | 0.000 |
| Intercept | 1604022.250 | 1 | 1604022.250 | 20865.330 | 0.000 |
| Photoperiod | 54843.250 | 3 | 18281.083 | 237.803 | 0.000 |
| Error | 922.500 | 12 | 76.875 |  |  |
| Total | 1659788.000 | 16 |  |  |  |
| Corrected Total | 55765.750 | 15 |  |  |  |

**Table 4b**: ANOVA table for shoot dry weight at harvesting stage.

| **Source** | **Type III Sum of Squares** | **df** | **Mean Square** | **F** | **Sig.** |
| --- | --- | --- | --- | --- | --- |
| Corrected Model | 75.451^a^ | 3 | 25.150 | 16.955 | 0.000 |
| Intercept | 1944.149 | 1 | 1944.149 | 1310.620 | 0.000 |
| Photoperiod | 75.451 | 3 | 25.150 | 16.955 | 0.000 |
| Error | 17.801 | 12 | 1.483 |  |  |
| Total | 2037.401 | 16 |  |  |  |
| Corrected Total | 93.252 | 15 |  |  |  |

**Table 4c**: ANOVA table for root fresh weight at harvesting stage.

| **Source** | **Type III Sum of Squares** | **df** | **Mean Square** | **F** | **Sig.** |
| --- | --- | --- | --- | --- | --- |
| Corrected Model | 84.063^a^ | 3 | 28.021 | 97.323 | 0.000 |
| Intercept | 2056.623 | 1 | 2056.623 | 7143.117 | 0.000 |
| Photoperiod | 84.063 | 3 | 28.021 | 97.323 | 0.000 |
| Error | 3.455 | 12 | 0.288 |  |  |
| Total | 2144.140 | 16 |  |  |  |
| Corrected Total | 87.518 | 15 |  |  |  |

**Table 4d**: ANOVA table for root dry weight at harvesting stage.

| **Source** | **Type III Sum of Squares** | **df** | **Mean Square** | **F** | **Sig.** |
| --- | --- | --- | --- | --- | --- |
| Corrected Model | .256^a^ | 3 | 0.085 | 14.236 | 0.000 |
| Intercept | 4.358 | 1 | 4.358 | 728.046 | 0.000 |
| Photoperiod | 0.256 | 3 | 0.085 | 14.236 | 0.000 |
| Error | 0.072 | 12 | 0.006 |  |  |
| Total | 4.685 | 16 |  |  |  |
| Corrected Total | 0.327 | 15 |  |  |  |

**Table 5**: ANOVA table for number of leaves at harvesting stage.

| **Source** | **Type III Sum of Squares** | **df** | **Mean Square** | **F** | **Sig.** |
| --- | --- | --- | --- | --- | --- |
| Corrected Model | 37.500^a^ | 3 | 12.500 | 6.977 | 0.006 |
| Intercept | 7225.000 | 1 | 7225.000 | 4032.558 | 0.000 |
| Photoperiod | 37.500 | 3 | 12.500 | 6.977 | 0.006 |
| Error | 21.500 | 12 | 1.792 |  |  |
| Total | 7284.000 | 16 |  |  |  |
| Corrected Total | 59.000 | 15 |  |  |  |

**Table 6**: ANOVA table for specific leaf area at harvesting stage.

| **Source** | **Type III Sum of Squares** | **df** | **Mean Square** | **F** | **Sig.** |
| --- | --- | --- | --- | --- | --- |
| Corrected Model | 240.762^a^ | 3 | 80.254 | 0.158 | 0.922 |
| Intercept | 1350615.866 | 1 | 1350615.866 | 2665.590 | 0.000 |
| Photoperiod | 240.762 | 3 | 80.254 | 0.158 | 0.922 |
| Error | 6080.227 | 12 | 506.686 |  |  |
| Total | 1356936.855 | 16 |  |  |  |
| Corrected Total | 6320.989 | 15 |  |  |  |

**Table 7**: ANOVA table for root shoot ratio at harvesting stage.

| **Source** | **Type III Sum of Squares** | **df** | **Mean Square** | **F** | **Sig.** |
| --- | --- | --- | --- | --- | --- |
| Corrected Model | .001^a^ | 3 | 0.000 | 4.957 | 0.018 |
| Intercept | 0.036 | 1 | 0.036 | 971.469 | 0.000 |
| Photoperiod | 0.001 | 3 | 0.000 | 4.957 | 0.018 |
| Error | 0.000 | 12 | 3.706E-05 |  |  |
| Total | 0.037 | 16 |  |  |  |
| Corrected Total | 0.001 | 15 |  |  |  |

**Table 8**: ANOVA table for plant height at harvesting stage

| **Source** | **Type III Sum of Squares** | **df** | **Mean Square** | **F** | **Sig.** |
| --- | --- | --- | --- | --- | --- |
| Corrected Model | 70.949^a^ | 3 | 23.650 | 9.123 | 0.002 |
| Intercept | 5485.254 | 1 | 5485.254 | 2115.859 | 0.000 |
| Photoperiod | 70.949 | 3 | 23.650 | 9.123 | 0.002 |
| Error | 31.109 | 12 | 2.592 |  |  |
| Total | 5587.313 | 16 |  |  |  |
| Corrected Total | 102.059 | 15 |  |  |  |

**Table 9**: ANOVA table for root length at harvesting stage

| **Source** | **Type III Sum of Squares** | **df** | **Mean Square** | **F** | **Sig.** |
| --- | --- | --- | --- | --- | --- |
| Corrected Model | 285.047^a^ | 3 | 95.016 | 1.920 | 0.180 |
| Intercept | 23908.891 | 1 | 23908.891 | 483.160 | 0.000 |
| Photoperiod | 285.047 | 3 | 95.016 | 1.920 | 0.180 |
| Error | 593.813 | 12 | 49.484 |  |  |
| Total | 24787.750 | 16 |  |  |  |
| Corrected Total | 878.859 | 15 |  |  |  |

**Table 10**: ANOVA table for leaf width at harvesting stage

| **Source** | **Type III Sum of Squares** | **df** | **Mean Square** | **F** | **Sig.** |
| --- | --- | --- | --- | --- | --- |
| Corrected Model | 66.172^a^ | 3 | 22.057 | 7.239 | 0.005 |
| Intercept | 6064.516 | 1 | 6064.516 | 1990.405 | 0.000 |
| Photoperiod | 66.172 | 3 | 22.057 | 7.239 | 0.005 |
| Error | 36.563 | 12 | 3.047 |  |  |
| Total | 6167.250 | 16 |  |  |  |
| Corrected Total | 102.734 | 15 |  |  |  |

**Physiological parameters**

**Table 11**: ANOVA table for Photosynthetic rate (Pn) in an iceberg lettuce

| **Source** | **Type III Sum of Squares** | **df** | **Mean Square** | **F** | **Sig.** |
| --- | --- | --- | --- | --- | --- |
| Corrected Model | 96.455^a^ | 5 | 19.291 | 49.852 | 0.0001 |
| Intercept | 3048.132 | 1 | 3048.132 | 7877.035 | 0.0001 |
| Photoperiod | 96.455 | 5 | 19.291 | 49.852 | 0.0001 |
| Error | 20.509 | 53 | 0.387 |  |  |
| Total | 3147.757 | 59 |  |  |  |
| Corrected Total | 116.965 | 58 |  |  |  |

**Table 12**: ANOVA table for Stomatal conductance in an iceberg lettuce

| **Source** | **Type III Sum of Squares** | **df** | **Mean Square** | **F** | **Sig.** |
| --- | --- | --- | --- | --- | --- |
| Corrected Model | .070^a^ | 5 | 0.014 | 39.697 | 0.0001 |
| Intercept | 1.131 | 1 | 1.131 | 3199.364 | 0.0001 |
| Photoperiod | 0.070 | 5 | 0.014 | 39.697 | 0.0001 |
| Error | 0.019 | 53 | 0.000 |  |  |
| Total | 1.224 | 59 |  |  |  |
| Corrected Total | 0.089 | 58 |  |  |  |

**Table 13**: ANOVA table for transpiration rate (E) in an iceberg lettuce

| **Source** | **Type III Sum of Squares** | **df** | **Mean Square** | **F** | **Sig.** |
| --- | --- | --- | --- | --- | --- |
| Corrected Model | 1.480^a^ | 5 | 0.296 | 3.989 | 0.004 |
| Intercept | 136.982 | 1 | 136.982 | 1845.562 | 0.000 |
| Photoperiod | 1.480 | 5 | 0.296 | 3.989 | 0.004 |
| Error | 3.934 | 53 | 0.074 |  |  |
| Total | 142.875 | 59 |  |  |  |
| Corrected Total | 5.414 | 58 |  |  |  |

**Table 14**: ANOVA table for the ratio of intercellular and ambient CO_2_ concentration (Ci/Ca) in an iceberg lettuce

| **Source** | **Type III Sum of Squares** | **df** | **Mean Square** | **F** | **Sig.** |
| --- | --- | --- | --- | --- | --- |
| Corrected Model | .190^a^ | 5 | 0.038 | 74.766 | 0.0001 |
| Intercept | 45.516 | 1 | 45.516 | 89668.043 | 0.0001 |
| Photoperiod | 0.190 | 5 | 0.038 | 74.766 | 0.0001 |
| Error | 0.027 | 53 | 0.001 |  |  |
| Total | 45.680 | 59 |  |  |  |
| Corrected Total | 0.217 | 58 |  |  |  |

**Table 15**: ANOVA table for intercellular CO_2_ concentration (Ci) in an iceberg lettuce

| **Source** | **Type III Sum of Squares** | **df** | **Mean Square** | **F** | **Sig.** |
| --- | --- | --- | --- | --- | --- |
| Corrected Model | 88188.388^a^ | 5 | 17637.678 | 147.666 | 0.0001 |
| Intercept | 9655239.750 | 1 | 9655239.750 | 80835.736 | 0.0001 |
| Photoperiod | 88188.388 | 5 | 17637.678 | 147.666 | 0.0001 |
| Error | 6330.464 | 53 | 119.443 |  |  |
| Total | 9733565.519 | 59 |  |  |  |
| Corrected Total | 94518.852 | 58 |  |  |  |

**Table 16**: ANOVA table for water use efficiency (Pn/E) in an iceberg lettuce

| **Source** | **Type III Sum of Squares** | **df** | **Mean Square** | **F** | **Sig.** |
| --- | --- | --- | --- | --- | --- |
| Corrected Model | 42.000^a^ | 5 | 8.400 | 24.565 | 0.0001 |
| Intercept | 1339.585 | 1 | 1339.585 | 3917.417 | 0.0001 |
| Photoperiod | 42.000 | 5 | 8.400 | 24.565 | 0.0001 |
| Error | 18.124 | 53 | 0.342 |  |  |
| Total | 1387.370 | 59 |  |  |  |
| Corrected Total | 60.124 | 58 |  |  |  |

**Qualitative parameters**

**Table 17**: ANOVA table for vitamin c content in an iceberg lettuce

| **Source** | **Type III Sum of Squares** | **df** | **Mean Square** | **F** | **Sig.** |
| --- | --- | --- | --- | --- | --- |
| Corrected Model | 30.541^a^ | 3 | 10.180 | 62.810 | 0.000 |
| Intercept | 1251.744 | 1 | 1251.744 | 7723.043 | 0.000 |
| Photoperiod | 30.541 | 3 | 10.180 | 62.810 | 0.000 |
| Error | 1.945 | 12 | 0.162 |  |  |
| Total | 1284.230 | 16 |  |  |  |
| Corrected Total | 32.486 | 15 |  |  |  |

**Table 18**: ANOVA table for total phenols in an iceberg lettuce

| **Source** | **Type III Sum of Squares** | **df** | **Mean Square** | **F** | **Sig.** |
| --- | --- | --- | --- | --- | --- |
| Corrected Model | 126.374^a^ | 3 | 42.125 | 36.414 | 0.000 |
| Intercept | 2538.648 | 1 | 2538.648 | 2194.496 | 0.000 |
| Photoperiod | 126.374 | 3 | 42.125 | 36.414 | 0.000 |
| Error | 13.882 | 12 | 1.157 |  |  |
| Total | 2678.904 | 16 |  |  |  |
| Corrected Total | 140.256 | 15 |  |  |  |

**Table 19**: ANOVA table for antioxidant capacity in an iceberg lettuce

| **Source** | **Type III Sum of Squares** | **df** | **Mean Square** | **F** | **Sig.** |
| --- | --- | --- | --- | --- | --- |
| Corrected Model | 7.956^a^ | 3 | 2.652 | 21.628 | 0.000 |
| Intercept | 957.903 | 1 | 957.903 | 7812.438 | 0.000 |
| Photoperiod | 7.956 | 3 | 2.652 | 21.628 | 0.000 |
| Error | 1.471 | 12 | 0.123 |  |  |
| Total | 967.329 | 16 |  |  |  |
| Corrected Total | 9.427 | 15 |  |  |  |

**Resource use efficiency**

**Table 20**: ANOVA table for water use efficiency of an iceberg lettuce

| **Source** | **Type III Sum of Squares** | **df** | **Mean Square** | **F** | **Sig.** |
| --- | --- | --- | --- | --- | --- |
| Corrected Model | 2890.223^a^ | 3 | 963.408 | 271.119 | 0.000 |
| Intercept | 46095.017 | 1 | 46095.017 | 12971.928 | 0.000 |
| Photoperiod | 2890.223 | 3 | 963.408 | 271.119 | 0.000 |
| Error | 42.641 | 12 | 3.553 |  |  |
| Total | 49027.881 | 16 |  |  |  |
| Corrected Total | 2932.865 | 15 |  |  |  |

**Table 21**: ANOVA table for energy use efficiency (FW) of an iceberg lettuce

| **Source** | **Type III Sum of Squares** | **df** | **Mean Square** | **F** | **Sig.** |
| --- | --- | --- | --- | --- | --- |
| Corrected Model | 6756.426^a^ | 2 | 3378.213 | 213.035 | 0.000 |
| Intercept | 357751.520 | 1 | 357751.520 | 22560.343 | 0.000 |
| Photoperiod | 6756.426 | 2 | 3378.213 | 213.035 | 0.000 |
| Error | 142.718 | 9 | 15.858 |  |  |
| Total | 364650.664 | 12 |  |  |  |
| Corrected Total | 6899.144 | 11 |  |  |  |

**Table 22**: ANOVA table for energy use efficiency (DW) of an iceberg lettuce

| **Source** | **Type III Sum of Squares** | **df** | **Mean Square** | **F** | **Sig.** |
| --- | --- | --- | --- | --- | --- |
| Corrected Model | 3.668^a^ | 2 | 1.834 | 5.778 | 0.024 |
| Intercept | 430.561 | 1 | 430.561 | 1356.549 | 0.000 |
| Photoperiod | 3.668 | 2 | 1.834 | 5.778 | 0.024 |
| Error | 2.857 | 9 | 0.317 |  |  |
| Total | 437.085 | 12 |  |  |  |
| Corrected Total | 6.524 | 11 |  |  |  |

**Table. 23** Energy requirement for different components of VHS

| **DLI (mol m^-2^ d^-1^) / Photoperiod(h)** | **Energy consumption** | | | | | | |
| --- | --- | --- | --- | --- | --- | --- | --- |
|  | LED (W) | Pump (W) | Aerator pump (W) | Total (w) | kWh | kWh/day | 45 days |
| **8.34/12** | 76.95 | 20.79 | 2.81 | 100.55 | 0.10055 | 1.20 | 54.30 |
| **11.5/16** | 76.95 | 20.79 | 2.81 | 100.55 | 0.10055 | 1.60 | 72.40 |
| **14.4/20** | 76.95 | 20.79 | 2.81 | 100.55 | 0.10055 | 2.01 | 90.50 |
